# Supplementary material for: Full-field thermal imaging of quasiballistic crosstalk reduction in nanoscale devices
Source: Nat Commun. 2018 Jan 17;9:255. doi: 10.1038/s41467-017-02652-4 (PMC5772674; doi:10.1038/s41467-017-02652-4)
Supplement: Supplementary file 1 — Supplementary Information [file 41467_2017_2652_MOESM1_ESM.pdf]

Supplementary Information

1. Thermoreflectance thermal imaging.

The principle of thermoreflectance (TR) thermal imaging is shown in Supplementary Figure 1a. For quasi steady-state measurement, we used 4-bucekt technique with a low-frequency (7.5 Hz) sinusoidal electrical current [1,2]. A constant LED light is illuminated on the device under test (DUT). Typically, 530nm illumination wavelength is used for gold samples. The reflected light from the DUT is captured by a CCD camera. From the change in reflectivity and using the calibrated coefficient of thermoreflectance (C_{TR}) of the material under study at the wavelength of the light, we measure the temperature of the device ($\Delta T=1/C_{TR}$ $(\Delta R/R_0)$). The calibrated C_{TR} for gold at 530nm under 100x objective lens is about -2.3×10^{-4} K^{-1}. The calibration is done on each device using temperature dependent current-voltage measurements (IVT). Average temperature of heater lines at different current is obtained using the IVT. By comparing the average temperature of the heater lines measured by IVT with that obtained by TR imaging, the C_{TR} is extracted. The results are also confirmed by independent calibration procedure explained elsewhere [3]. Supplementary Figure 1b shows the schematic for transient measurement procedure. We excite the DUT with an electrical current pulse with a given duty cycles. In the main text, the temperature evolution in response to a 1μs electrical pulse with 10% duty cycle is plotted. The LED light is then illuminated on the sample at the desired delay and the reflected light at that time is captured (hot frame). From the change in reflectivity, with respect to the reflectivity when there is no electrical bias (the cold frame), the temperature at each time step can be obtained ($\Delta T=1/C_{TR}$ $(R_{hot}$ - $R_{cold})/R_{cold}$). It is shown in supplementary reference [4] and also in Ziabari, et al. (manuscript in preparation) that TR imaging can be used for imaging devices down to diffraction limit (200-300 nm).

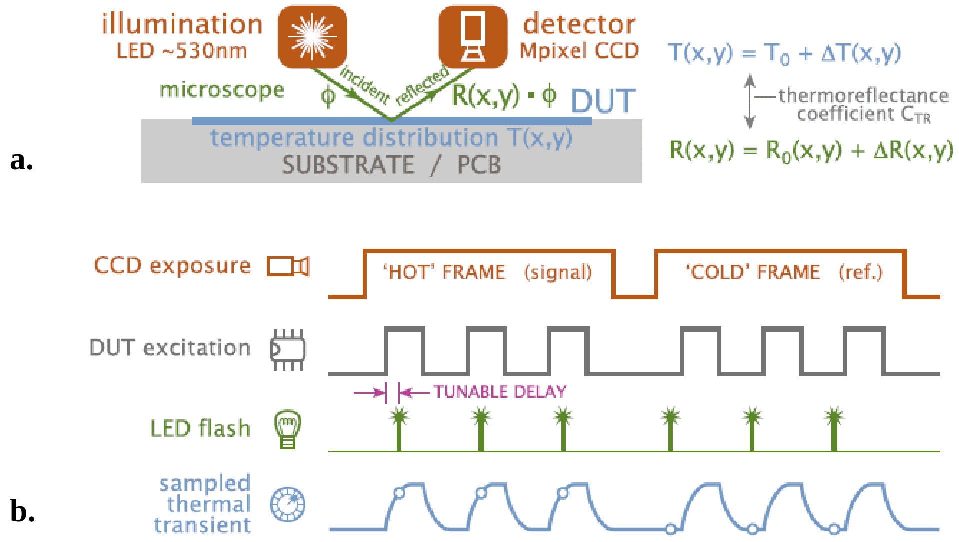

Supplementary Figure 1. Principle of thermoreflectance thermal imaging[1]. Reprint with permission from AIP publishing LLC. a. General principle; b. Transient thermoreflectance thermal imaging procedure.

2. 3ω and TDTR Techniques

The principles of 3ω and TDTR techniques have already been described in literature[5,6]. Here, we will present some of the raw data obtained by these techniques.

Supplementary Figure 2a and b shows the results obtained from 3ω and fitting model. The extracted nominal thermal conductivity of InGaAs, InP are 5.35$Wm^{-1}K^{-1}$ and 69.9 $Wm^{-1}K^{-1}$, which used for simulation of larger device sizes. The effective interface thermal conductivity of 20nm Al_2O_3 is 0.63$Wm^{-1}K^{-1}$. InGaAs thermal conductivity and effective interface thermal conductivity are also obtained using TDTR to be 5.4±0.4, and 0.65±0.07, respectively.

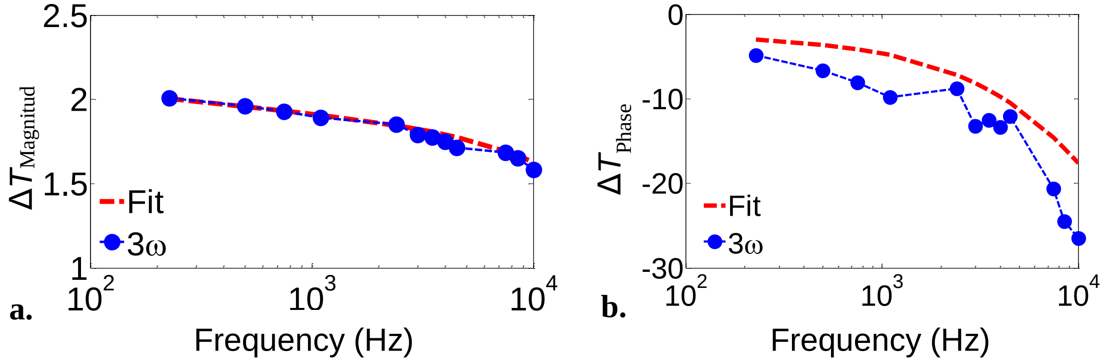

Supplementary Figure 2. 3ω results. The fitted line suggests a thermal conductivity of 5.35Wm^{-1}K^{-1} and 69.85 Wm^{-1}K^{-1} for InGaAs, InP, respectively. The effective interface thermal conductivity of 20nm Al_2O_3 is 0.63 Wm^{-1}K^{-1}. a. Magnitude; b. Phase.

3. Gold properties

The measured properties for gold are shown in Supplementary Figure 3a and b. Gold's electrical resistivity, thermal conductivity, coefficient of temperature dependence of resistivity, and coefficient of thermoreflectance, are plotted against device widths.

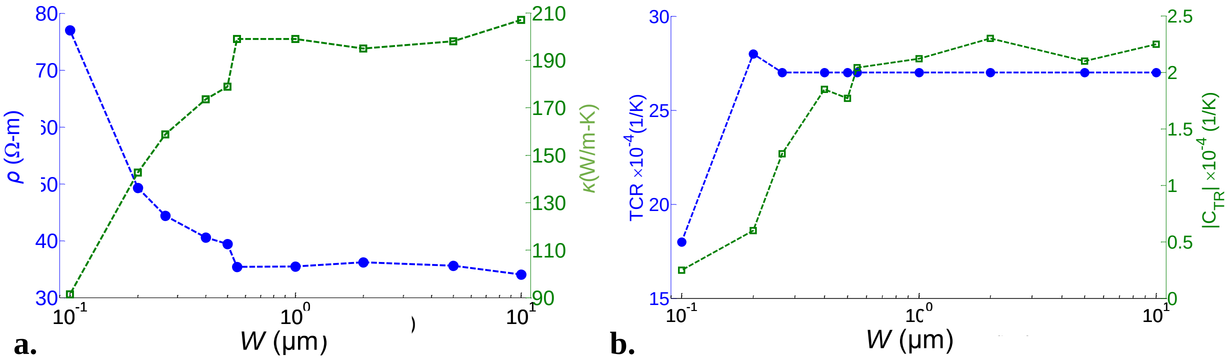

Supplementary Figure 3. a. Electrical resistivity (ρ) and thermal conductivity (κ) of gold measured for different widths. b. Coefficient of temperature dependence of resistivity (TCR), and coefficient of thermoreflectance (C_{TR}) measured for gold at different heater line widths.

4. Temperature distribution

As it is shown in Figure 3 in the main text, the temperature distribution on the substrate next to heater line and the temperature distribution on a thermometer line within few microns of the source cannot be predicted by the same InGaAs thermal conductivity. This is further demonstrated in Supplementary Figure 4.

Supplementary Figures 4a-e show the temperature profiles of heater lines fabricated within few microns of a thermometer line. Electrical current was sent through the heater line to change its temperature and the neighbouring line serves as a thermometer. The dimensions of the heater lines as well as the thermometer lines, measured by scanning electron microscopy (SEM), are given on top of each device temperature map. Colour scales are adjusted in Supplementary Figures 4a-e to clearly show heat spreading in the substrate and through the thermometer lines. The temperature cross sections of different devices along A-A', perpendicular to the lines in direction of y-axis, are plotted in Supplementary Figures 4f-j. A ~5.4 Wm^{-1}K^{-1} for 10 μm device, and a 4.5 Wm^{-1}K^{-1} for the 265nm device were used

in the FEM model, which corresponds to the same number shown in Figure 3c (main text). It is evident that as the width of the heater lines decreases, the deviation of the temperature distribution on the thermometer lines next to the device from the modified Fourier model increases. In fact, this effect is magnified in Supplementary Figures 4k-o. At 265nm, the average temperature on the thermometer line is ~80% lower than that of predicted by modified Fourier model. Hence, Supplementary Figure 4 illustrates that although by using an apparent thermal conductivity in the Fourier model we can predict and fit the experimental results, the full distribution of temperature profile cannot be predicted.

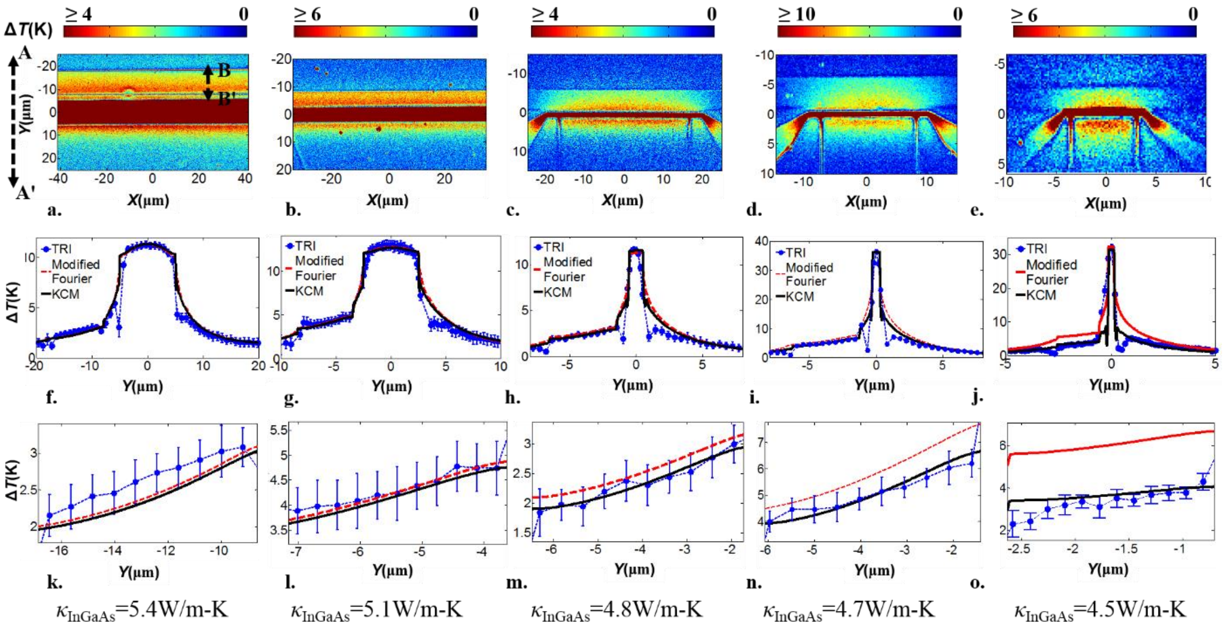

Supplementary Figure 4. Study of temperature profiles along the heater line, thermometer line and the substrate. Temperature profiles of two heater lines in parallel. One of the heater lines acts as the heater and the other act as a thermometer. a. A 10μm heater line, 10μm thermometer line, gap size of 3μm; b. A 5μm heater line, 5μm thermometer line, gap size of 1μm; c. A 1μm heater line, 5μm thermometer line, gap size of 1μm; d. A 550nm heater line, 5μm thermometer line, gap size of 1 μm; e. A 265nm heater line, 2μm thermometer line, gap size of 480nm. The aspect ratios (length/width) is 40 (30 in the case of 10 μm heater line). The colour scales are adjusted in the profiles to clearly show heat spreading in the substrate and through the thermometer lines. f-j. Temperature cross section across the y-axis. In the modified Fourier model (red curves), the Thermal conductivity of the substrate is modified so that the modelling temperature profile at the top agrees with experimental results: f. 5.4 $Wm^{-1}K^{-1}$; g. 5.1 $Wm^{-1}K^{-1}$; h. 4.8 $Wm^{-1}K^{-1}$; i. 4.7 $Wm^{-1}K^{-1}$; j. 4.5 $Wm^{-1}K^{-1}$. The black curve shows the KCM results assuming $l=150nm$. Blur dots are the experimental results averaged over few neighbouring pixels on top of the heater line. Each data point is obtained by averaging few neighbouring pixels along horizontal axis in temperature profiles, and the errorbars are the standard deviation of those pixels. k-o. As the size of heater line decreases, the temperature near the heat source in the experiment deviates significantly from that obtained from the modified Fourier. KCM results match well the full temperature profile.

To fit the steady-state temperature distribution on top of the heater line and its surrounding using a Fourier model, one needs to use an anisotropic thermal conductivity for the InGaAs thin film in the ANSYS Finite Element Model. First, a semi-analytical model was developed to serve as an optimization tool to find the InGaAs thermal conductivity that best fit experimental results as the size of heater lines decrease. All other material properties were input based on the independent electrical and thermal measurement. The extracted thermal conductivity was then input to the full 3D FEM model to obtain full temperature distribution of heater and thermometer lines. From this modelling, the entire temperature distribution for the 265nm line was fitted with a cross-plane thermal conductivity of $10Wm^{-1}K^{-1}$ and in-

plane value of ~1.5$Wm^{-1}K^{-1}$. Not only these values are quite surprising, but also they are not consistent for different heat sources. For each measurement, a new pair of cross- and in-plane thermal conductivities must be obtained. Reduced apparent thermal conductivity has been attributed to phonons with long mean free path not contributing to heat transport. It is puzzling why this would give an effective cross-plane thermal conductivity that is 220% higher than the bulk value but in-plane value that is 300% lower for an isotropic material such as InGaAs. Also, as it is shown in supplementary note 5, even an anisotropic thermal conductivity cannot explain both steady-state and transient experimental results.

It is worth mentioning that Wilson and Cahill [7] used a combination of anisotropic thermal conductivity and modified boundary conditions at the metal/semiconductor interface to explain the observed frequency-dependence and beam size effects in ultrafast TDTR measurements. Hua et al. [8] also pointed out that one needs to go beyond a simple interface thermal boundary resistance between the metal and the semiconductor and consider the spectral phonon transmission coefficient to explain the rich TDTR data for different alloys (e.g. SiGe) as well as non-alloys (e.g. Si). These approaches are discussed in the next section.

5. Comparison with previous works: study of anisotropic Fourier model, TBR effect and tempered Lévy superdiffusion

This work presents a complete 2D temperature map of a nanoheater on a surface with submicron precision. In most previous studies, thermal responses were obtained using only single point temperature information or averaged laser probe.

5.1) Using a bulk effective thermal conductivity of the substrate:

Minnich et al.[9] used a phonon diffusive – ballistic transition to explain their results. The final effect is an effective reduction of the substrate thermal conductivity. This approach was used because their experimental data consisted only in the temperature decay of the heater and consequently only effective information at the source was obtained. Several other authors made the same observation using TDTR technique which are listed in the main text. The temperature profile of a 265nm heater line along with temperature cross sections are shown in Supplementary Figure 5. The red dots are the experimental results in this figure and the dash lines are the FEM (Fourier model results). Akin to the prior works in literature, using a reduced thermal conductivity of 4.5$Wm^{-1}k^{-1}$ for InGaAs (black line) we can fit the heater temperature at the top as shown in Supplementary Figure 5b, and c. However, we observe an overprediction of temperature on the neighbouring thermometer line meaning we cannot explain the entire temperature profile with a reduced thermal conductivity. On the other hand, we can use a larger thermal conductivity of 8 $Wm^{-1}k^{-1}$ to match the tail of temperature distribution on the thermometer. Using that value for thermal conductivity, however, we cannot match the temperature profile at the top of the device (blue line in Supplementary Figure 5b and c). In general, changing to an effective thermal conductivity we can locally match the average temperature as was done in previous studies, but there is not a single effective thermal conductivity that matches the entire temperature profile.

5.2) Using an anisotropic thermal conductivity.

A similar (but not the same) observation was made by Wilson and Cahill[7]. In their beam-offset TDTR setup an averaged measure of the temperature on the heat source and near the heated regions was obtained. A lower temperature than the one predicted by the effective Fourier was observed. This setup offers the possibility to fit to two points on the profile, using an anisotropic thermal conductivity. The green curve in Supplementary Figure 5b and c show the results. Although the obtained curve is better than the single parameter approach, there are few issues with this approach:

1. It is hard to justify an anisotropic thermal conductivity of 1.5 $Wm^{-1}K^{-1}$ in-plane and 10 $Wm^{-1}K^{-1}$ cross-plane for five microns thick InGaAs (cross plane value is 50% larger than TDTR data on the same sample and in-plane value is 400% smaller).
2. For each nanoheater device size a new pair of thermal conductivity values is required to fit the temperature profiles.

3. There are samples for which that we could not find a pair of anisotropic thermal conductivity to fit the entire temperature profile
4. Even for the measurement in Supplementary Figure 5 the thermal conductivity pair does not match the entire temperature profile and disagreement on the tails still exist

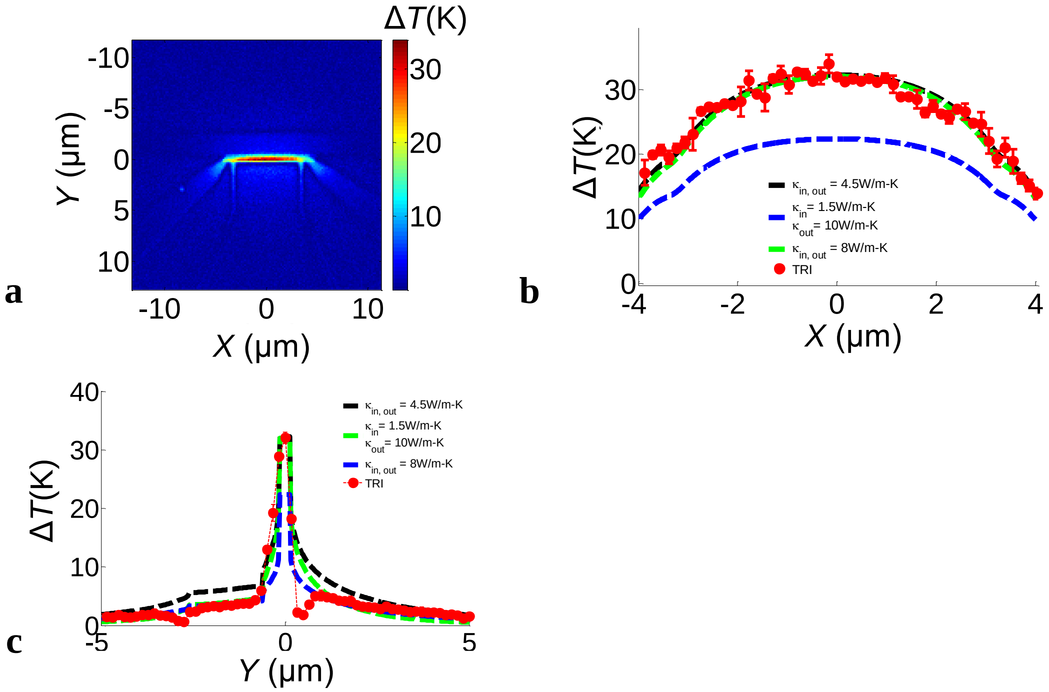

Supplementary Figure 5. Modified Fourier model with isotropic and anisotropic conductivity are not sufficient to explain the experimental results. a. Temperature profile of a 265nm heater line. b. cross section along the heater line. red dots are the experimental results. black and blue lines are Fourier simulation with isotropic thermal conductivity of 4.5Wm^{-1}K^{-1} and 8 Wm^{-1}K^{-1}. Green line is the Fourier results with anisotropic thermal conductivity (10 Wm^{-1}K^{-1} cross-plane and 1.5 Wm^{-1}K^{-1} in-plane). Each data point is obtained by averaging few neighbouring pixels along horizontal axis (image a), and the errorbars are the standard deviation of those pixels. c. Vertical cross-section compares the experimental and FEM results. Figures b and c shows that a lower conductivity can fit the data at top of the heater line and a larger conductivity could match the data on the tail, however to match the full distribution we needed anisotropic thermal conductivity in the Fourier model which is hard to justify for 5μm thick film. Additionally, the same combination of anisotropic thermal conductivity doesn't fit all the experimental data and only applies at 265 nm.

5.3) Using a Thermal boundary resistance.

Several works have interpreted deviations from Fourier diffusion in terms of an effectively increased Thermal Boundary Resistance (TBR) between the heat source and the substrate. For example, Siemens et al[10] used a constant TBR along with a modified thermal conductivity to explain their experimental results. Here we took the same approach and used a separate TBR in addition to oxide and substrate layers to explain the experimental results. experimental results are summarized in supplementary Table 1 and Supplementary Figure 6. Supplementary Figure 6 shows the experimental results (blue dots) for 265nm heater line and modelling results with different pairs of TBR and thermal conductivity. Using a TBR of 9 nK·m^{2}W^{-1} (dark red line) we can fit the heater temperature but we obtain poor predictions for the rest of the points. If we also change the thermal conductivity using a two-parameter approach to fit to heater and temperature (red line), we can get a curve which is similar to the hydrodynamic (KCM) approach.

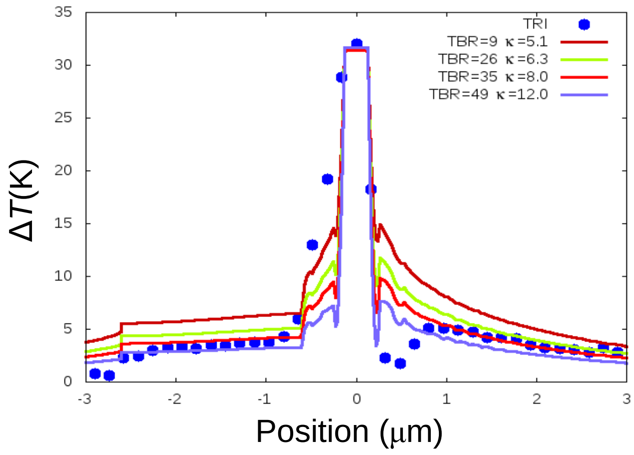

Supplementary Figure 6. Comparison between experimental and numerical results for horizontal temperature cross section along 265nm heater line. Blue dots are experimental results. Different lines correspond to different pairs of TBR and thermal conductivity. The pairs are changed until the best fit between numerical and the experimental results are obtained.

If we repeat this procedure for the rest of the lines we get the results summarized in the supplementary Table 1 as the best fitting results. Using this approach has doubled the number of fitting parameters, the thermal conductivity and the value of the TBR. A distinct pair of values is needed to fit observations at each device dimension. In addition, the main drawback of this approach is that the magnitude of the obtained TBRs are very large compared to the TDTR measurements and the known values of TBR models such as Diffuse or Acoustic Mismatch in the order of some nK·m^{2}W^{-1}.

Supplementary Table 1. The best pairs of TBR and thermal conductivity (κ) to fit the experimental results at different width

Line	200 nm	400 nm	500 nm	5 μm	10 μm
κ (W/m·K)	8.1	5.6	6.1	5.3	5.3
TBR (nK·m^2/W)	38.1	40.6	33.7	14.7	9.2

5.4) Analysis based on superdiffusive Lévy transport

Here we analyse the heater line configuration using a 3D tempered Lévy framework. As summarised below, this approach readily yields the correct trend at the source (increased self-heating and corresponding higher temperature at the heater line), but is unable to reproduce the lower thermal crosstalk (and corresponding lower temperature) measured at the nearby thermometer.

The superdiffusive framework has been extended to multidimensional heat flow by one of the authors recently[11] The evolution of volumetric thermal energy density P in an infinite medium can be described in terms of an isotropic stochastic process. In Fourier-Laplace domain the single pulse response takes the form

$$\mathrm{P}(\|\vec{\xi}\|, s) = \frac{1}{s + \psi(\|\vec{\xi}\|)}$$ Supplementary equation 1

where $\vec{\xi}$ denotes the spatial frequency vector and s is the Laplace variable.

3D transport in semiconductor alloys is found to obey a tempered Lévy process.

$$\psi(\zeta) = \frac{D\zeta^2}{(1 + r_{LF}^2\zeta^2)^{1-\frac{\alpha}{2}}} \qquad where\ \zeta \equiv \sqrt{\zeta_x^2 + \zeta_y^2 + \zeta_z^2}$$ Supplementary equation 2

At long length scales ($\zeta r_{LF} \ll 1$) this converges to regular diffusion $\psi \simeq D\zeta^2$ with bulk (Fourier) diffusivity D while short length scales ($\zeta r_{LF} \gg 1$) exhibit pure Lévy dynamics $\psi \simeq D_\alpha \zeta^\alpha$ with superdiffusion exponent $1 < \alpha < 2$ and fractional diffusivity $D_{alpha} \equiv D / r_{LF}^{2-\alpha}$ (unit $m^\alpha s^{-1}$). The transition between the two asymptotic regimes takes place over characteristic length scale r_{LF}. Tempered Lévy analysis of TDTR experiments on an InGaAs sample produced[11]

$$\alpha = 1.71\,, r_{LF} = 550\mathrm{nm}$$

We adopted these values alongside bulk diffusivity $D = 3.55\mathrm{mm}^2 s^{-1}$ (conductivity $\kappa = 5.5\mathrm{Wm}^{-1}\mathrm{K}^{-1}$) in the simulations that follow. We note that the sample used in the present work has a larger film thickness than the one previously measured with TDTR and thus likely possesses slightly different parameter values, but as these deviations do not affect the key outcome in any way they can be safely ignored.

To compute the thermal profile induced by a heater line, we first obtain the steady-state (s = 0) Green's function in real space through 3D Fourier inversion:

$$G(r) = \frac{1}{2\pi^2}\int_0^\infty \zeta^2 \frac{1}{\psi(\zeta)} j_0(\zeta r) d\zeta \qquad \text{where} \quad j_0(u) = \frac{sin(u)}{u}$$ Supplementary equation 3

The integral must be evaluated numerically. The result, which as expected transitions between pure Lévy and Fourier asymptotes (both of which are available in closed form), can be described compactly as

$$G(r) \simeq \frac{\left[1 + \left(\frac{r_0}{r}\right)^m\right]^{\frac{2-\alpha}{m}}}{4\pi D r} \quad , r_0 = 2\left[\frac{\Gamma\left(\frac{3-\alpha}{2}\right)}{\sqrt{\pi}\,\Gamma\left(\frac{\alpha}{2}\right)}\right]^{\frac{1}{2-\alpha}} . r_{LF}$$ Supplementary equation 4

For the InGaAs parameters listed earlier, m =1.62 provides the best fit (deviations ≤ 1.5%)
Finally, we obtain the lateral thermal profile induced by a rectangular heater line W × L through superposition:

$$\mathrm{P(x)} = \int_{-\mathrm{w}/2}^{\mathrm{w}/2} \mathrm{dx'} \int_{-\mathrm{L}/2}^{\mathrm{L}/2} \mathrm{dy'} \mathrm{G}\left(\mathrm{r} = \sqrt{(\mathrm{x} - \mathrm{x'})^2 + \mathrm{y'}^2}\right)$$ Supplementary equation 5

We carry out the 2D integration numerically using simple weighted summation of G over an adaptive, logarithmically spaced *(x', y')* rectangular mesh.

In diffusive regime, the solution can be derived fully analytically:

$$P_{Fourier}(x) = \frac{W}{8\pi D}\left[(\chi - 1)\, ln\left(\frac{\sqrt{(\chi-1)^2+\beta^2}-\beta}{\sqrt{(\chi-1)^2+\beta^2}+\beta}\right) + (\chi + 1)\, ln\left(\frac{\sqrt{(\chi+1)^2+\beta^2}+\beta}{\sqrt{(\chi+1)^2+\beta^2}-\beta}\right) + 2\beta\, ln\left(\frac{\sqrt{(\chi-1)^2+\beta^2}-(\chi-1)}{\sqrt{(\chi+1)^2+\beta^2}-(\chi+1)}\right)\right]$$

Supplementary equation 6

Here, $\chi = \frac{2x}{W}$, $\beta = \frac{L}{W}$.

We set the line aspect ratio β*=40* for the simulations in accordance with the fabricated samples, and then compute profiles for a variety of heater ranging in widths from 0.1μm to 10μm. We validated our computation scheme by

observing excellent agreement between the numerical result for a tempered Lévy kernel with $\alpha = 1.999$ and the analytical Fourier solution (Supplementary Figure 7).

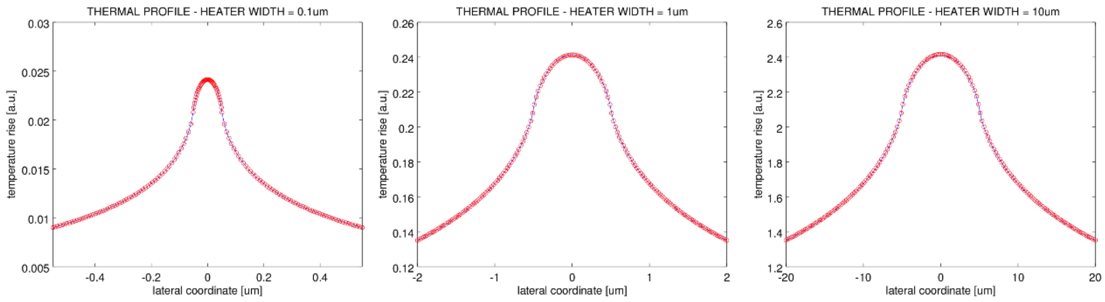

Supplementary Figure 7. Validation of integration scheme. Red circles: numerical result for tempered Lévy model with $\alpha = 1.999$; blue lines: analytical Fourier solution.

Supplementary Figure 8 shows the tempered Lévy results. Similar to the experiment, the junction temperatures (within the heater line) increasingly exceed conventional predictions as the heater line gets narrower.

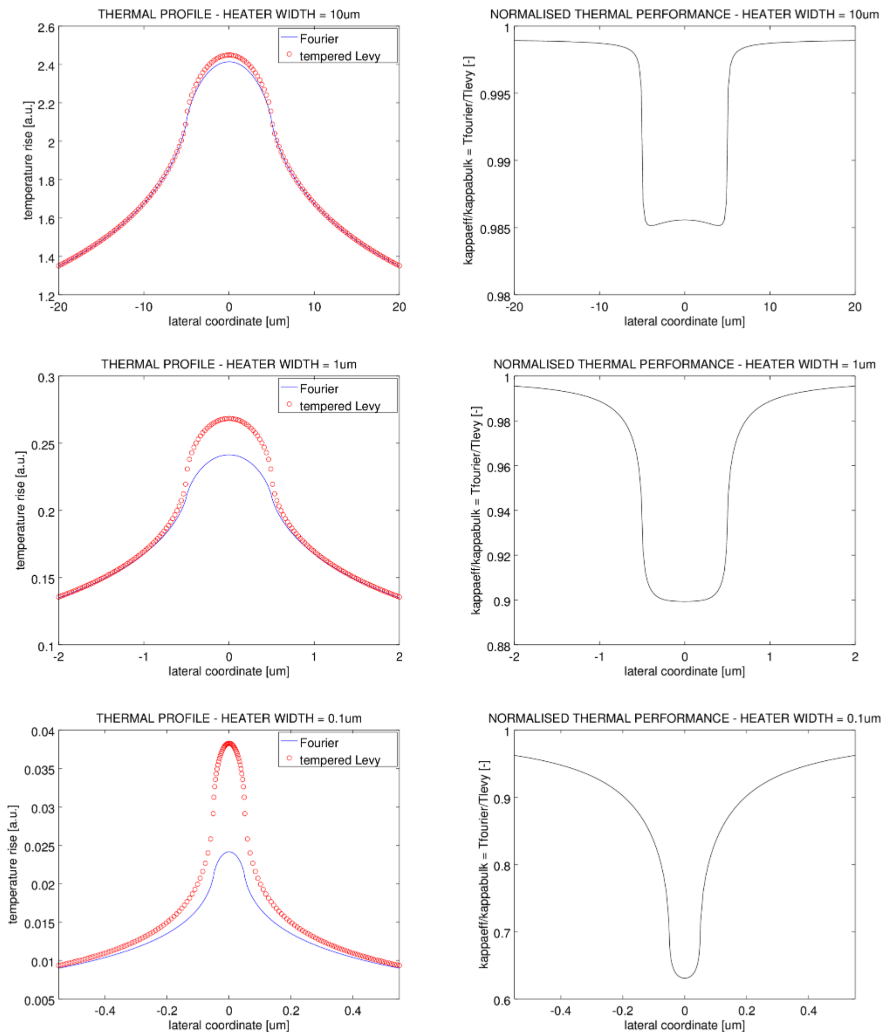

Supplementary Figure 8. Tempered Lévy heating profiles and associated effective thermal performance for heater widths of 100nm, 1μm and 10μm.

However, contrary to measurement, the tails of the profile never go below the diffusive solution, but always stay slightly above. In other words, the thermal crosstalk at a nearby thermometer (taken to be half a micron away from the line, i.e. $x = \frac{W}{2} + 0.5\mu m$) is either near nominal or even increased, but never reduced (Supplementary Figure 9).

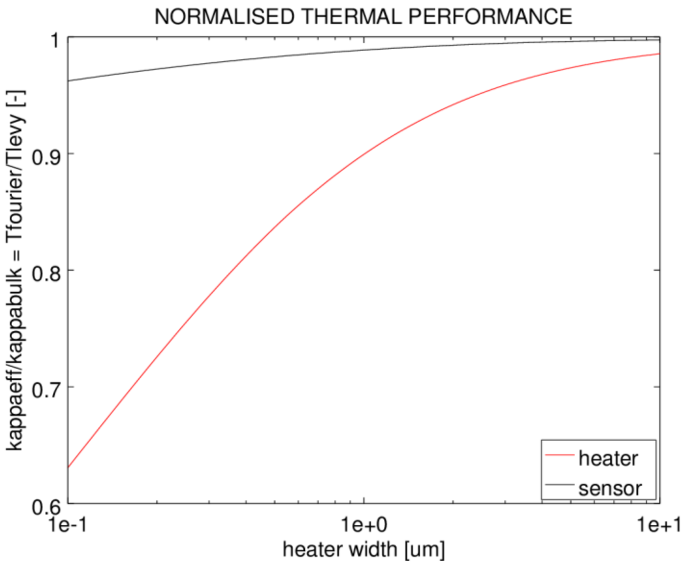

Supplementary Figure 9. Tempered Lévy simulation of effective thermal performance at the heater ($x = 0$) and adjacent thermometer half a micron away from the heater line ($x = \frac{W}{2} + 0.5\mu m$).}

Failure of the tempered Lévy model to reproduce the measured tail anomalies is a direct and inevitable consequence of the fact that the associated Green's function nowhere crosses over with the diffusive counterpart (the response in the asymptotic Lévy regime, where $G \sim \frac{1}{r^{3-\alpha}}$, always exceeds the Fourier one). One should note that this failure would equally occur in BTE analysis within the relaxation time approximation (RTA). Indeed, the rigorous single pulse response of the 1D RTA-BTE invariably displays impeded thermal transport that gradually recovers (but never trumps) diffusive behaviour[12]; mathematical symmetry[13] automatically conserves these traits in isotropic solutions of the multi-dimensional BTE.

Explaining the present measurement therefore clearly requires additional physics going beyond the current state-of-the-art RTA/superdiffusion formalisms. We should emphasize that the hydrodynamic model presented in the manuscript, can explain the reduced crosstalk near nanoheater sources due to hydrodynamic vorticities in the heat flow. We only need to fit the apparent thermal conductivity as the other parameter (nonlocal scale parameter) is equal for all the lines (l =150 nm). A unified description including both hydrodynamic heat equation and tempered Lévy is not currently available and it is the subject of future research.

6. Control Sample to validate the experiments

We fabricated an identical set of nanoheater lines on top of 3.5µm of SiO_2 and on silicon substrate to serve as control samples and validate techniques used in this work. We measured several devices. Supplementary Figure 10, summarize the results for 1µm, 530nm and 300nm device sizes. The experimental results for these devices are compared with the FEM results. We used 1.41$Wm^{-1}K^{-1}$ for oxide and 124 $Wm^{-1}K^{-1}$ for silicon thermal conductivity respectively, and 6.9 nKm^2W^{-1} for TBR between Au and SiO_2 in all the FEM simulations. The temperature profile both at the top of the heater line and on the neighbouring thermometer agrees with the modelling which suggest no non-diffusive effect exist in these samples and a Fourier diffusive model can explain the entire temperature profile for different device sizes in an amorphous oxide material.

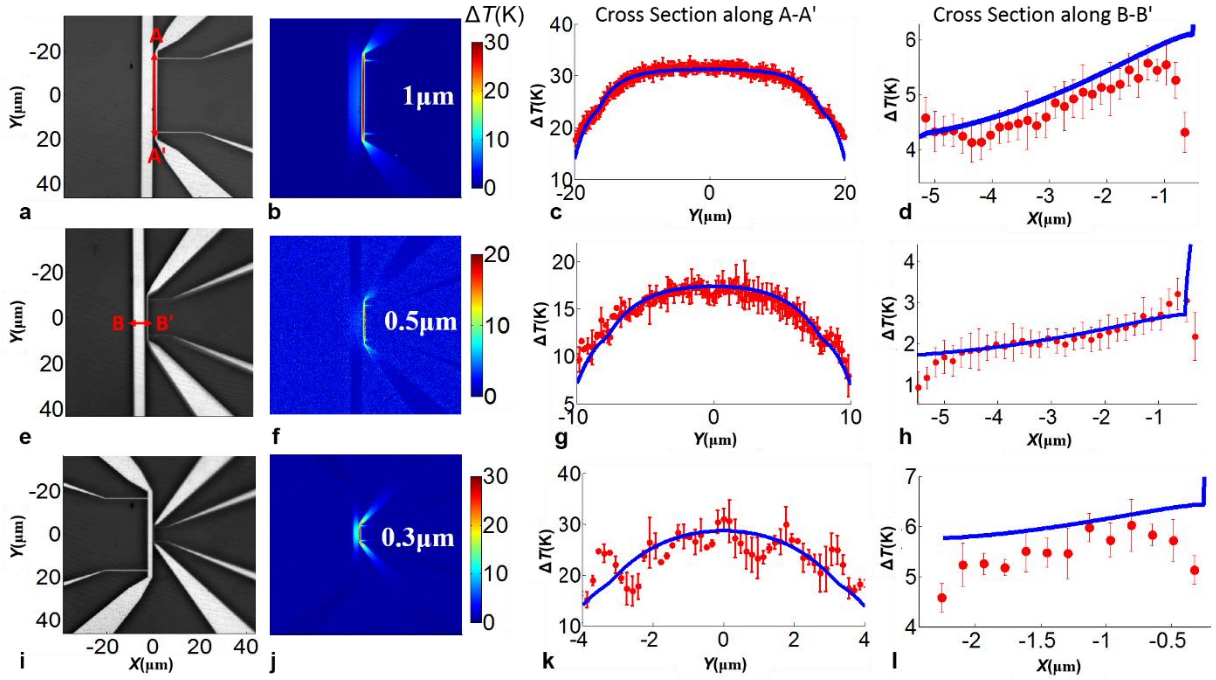

Supplementary Figure 10. Comparison between the experimental and modelling results on nanoheater lines on SiO_2/Si (3.5µm/500µm). a,e,i) optical images of 1µm, 530nm, and 300nm heater lines. b, f, j) Temperature profiles of the 1µm, 500nm, and 300nm heater lines. c, g, k) Vertical cross section of temperature profile along y direction (along A-A' shown in Figure a) on top of the heater line. d, h, l) Horizontal cross section along the x direction (along B-B' shown in Figure e) on top of the thermometer. The red dot are experimental results. Each data point is obtained by averaging few neighbouring pixels along vertical axis (images b, f, and j), and the errorbars are the standard deviation of those pixels. The blue line is the modelling results. The temperature profiles both on top of the heater line and the neighbouring thermometer line agrees very well between experiment and modelling. The same conductivity values were used in all the modelling results (1.41 $Wm^{-1}K^{-1}$ for oxide and 124 $Wm^{-1}K^{-1}$ for silicon, and 6.9 nKm^2W^{-1} for TBR between Au and SiO_2). These results show that the transport in oxide is diffusive and a Fourier diffusive model can simply explain the entire temperature profile.

7. Impact of Wavelength of the Light on TR Imaging

An example is shown in Supplementary Figure 11. Supplementary Figure 11a and b show the optical and temperature profile images of a 265nm heater line that is within 500nm of a 2µm thermometer line. The vertical cross section is plotted in Supplementary Figure 11c. Blue, green and red dots are the temperature measurements using 455nm, 530nm and 660nm LED lights, respectively. The black line is the FEM result using the effective thermal conductivity of 4.5$Wm^{-1}K^{-1}$ for InGaAs to match the temperature profile at the top. Supplementary Figure 11d shows the expanded view of the temperature cross section on the thermometer line. All the results producing the same observation that the temperature reduced on the thermometer by a factor of ~2x compared to the Fourier predictions suggesting that the observation is not an optical artefact.

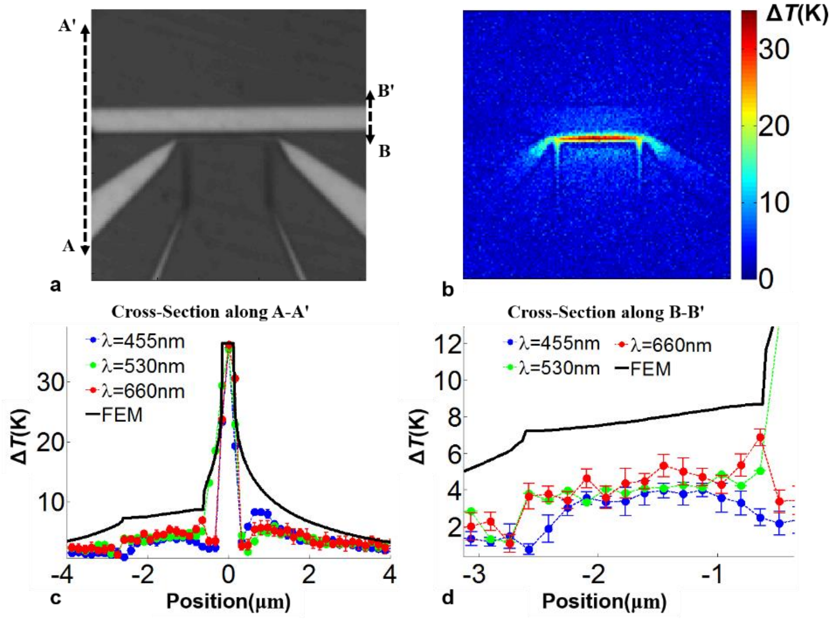

Supplementary Figure 11. Impact of changing the wavelength of the LED light in TR imaging on the measured temperature of the 265nm heater line. a. Optical Image of a 265nm heater line along with a 2μm thermometer line. b. Temperature profile at *I*=6.2mA. c. vertical Cross Section along a 265nm at 3differet wavelengths (455nm (blue), 530nm (green), 660nm (red)) compared with FEM (black line). d. Expanded view of the temperature profile cross section on the thermometer is plotted. It can be seen it is off by about 50%. Each data point is obtained by averaging few neighbouring pixels along horizontal axis (in image b), and the errorbars are the standard deviation of those pixels.

8. Impact of Temperature Dependence of InGaAs Thermal Conductivity

Temperature-dependent thermal conductivity of InGaAs is negligible in the range of localized heating studied in this work. While the average temperature change at the top of the metal is higher, the temperature on the substrate is much lower and temperature dependence of InGaAs would not play a role. For example, in Figure 3b, while the temperature change at the top is about 35K, the tail is about 4K. The temperature of the InGaAs does not increase beyond 10K. Further, the temperature-dependent thermal conductivity of InGaAs in 300-340K range is negligible (less than 5% change based on the TDTR data).

In addition, Supplementary Figure 12 below shows the temperature cross section of the a 10μm heater line obtained from TR measurements at two different power levels of *P1*=57mW and *P2*=79.1mW. The temperature cross section at power *P2* is shown in blue. If we normalize this temperature cross section with the ratio of *P1/P2* we get the green curve which agrees within 1% with the temperature cross section at *P1* (Red curve). Temperature agreements at the top and the tail suggest that high temperature of the metal doesn't have significant effect on InGaAs and temperature dependent of InGaAs does not play a significant role.

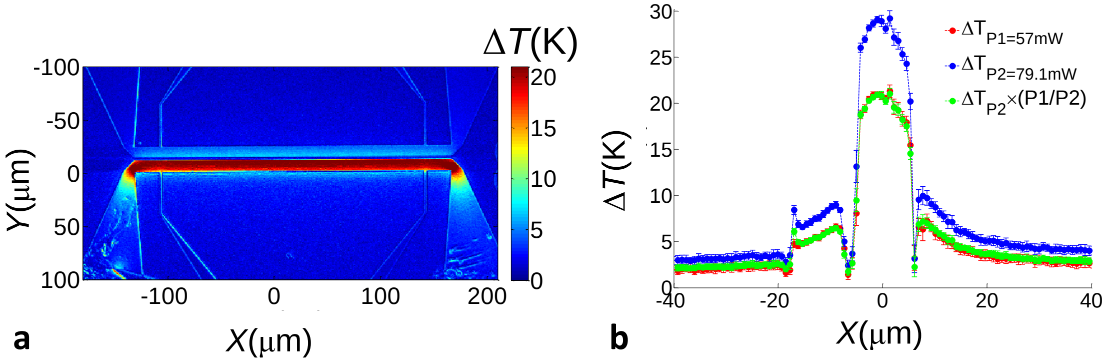

Supplementary Figure 12. Temperature profile linearly scales with the input power density. a. Temperature profile of a 10µm heater line. b. The vertical temperature cross section obtained from TR measurements at two different power level of *P1*=57mW (red curve) and *P2*=79.1mW (blue curve) is plotted. By normalizing the blue curve with the ratio of *P1/P2* we get the green curve which is almost the same curve as is the red curve and shows the large temperature change at top of the heater line doesn't have any effect on the neighbouring devices where the temperature change is much smaller than the top. Each data point is obtained by averaging few neighbouring pixels along horizontal axis (image a), and the errorbars are the standard deviation of those pixels.

9. Extent of the Hydrodynamic Flow Effect

Supplementary Figure 13a and b show a comparison between heat flux and temperature gradient in KCM and FEM models. The misalignment between heat flux and temperature gradient near the top surface in KCM model extends beyond the neighbourhood of the heat source and reaches below the thermometer region. This in turn illustrate that the impact of the vorticity near heat source can be captured with thermal imaging and electrical measurements at the thermometer. This is evident in all thermal profiles for heaters that are smaller than ~0.5 microns in width in the thermometer region ~1-2 microns away (see Figure 3 and supplementary Figure 4).

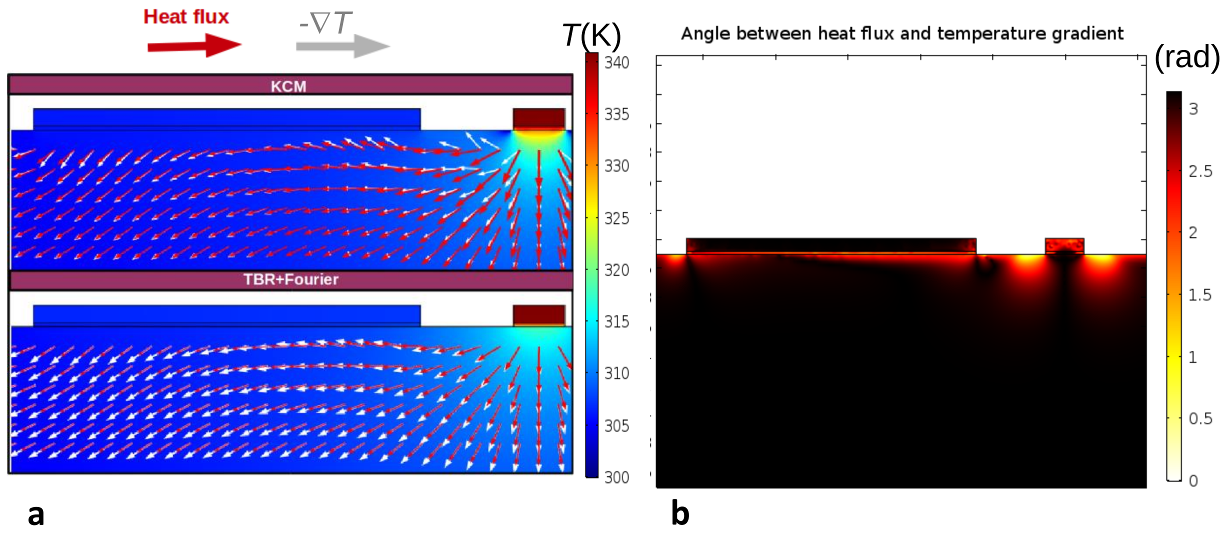

Supplementary Figure 13. a. The Comparison between heat flux and -∇T (negative gradient of temperature) vectors in KCM and Fourier models. b. The misalignment (angle difference) between heat flux and temperature gradient vectors adjacent to small sources. It is clear that the misalignment near the top surface in KCM model extends beyond the neighbourhood of the heat source and affects the thermometer region. This in turn illustrate that vorticity is not limited the region near heat source and can be captured with thermal imaging and electrical measurements by measuring the temperature of the thermometer.

Supplementary References

1. Vermeersch, B., Bahk, J.-H., Christofferson, J. & Shakouri, A. Thermoreflectance imaging of sub 100 ns pulsed cooling in high-speed thermoelectric microcoolers. *J. Appl. Phys.* **113,** 104502-1–8 (2013).

2. Favaloro, T. *et al.* Direct Observation of Nanoscale Peltier and Joule E ff ects at Metal − Insulator Domain Walls in Vanadium Dioxide Nanobeams. *Nano Lett.* **14,** 2394–2400 (2014).

3. Favaloro, T., Bahk, J.-H. & Shakouri, A. Characterization of the temperature dependence of the thermoreflectance coefficient for conductive thin films. *Rev. Sci. Instrum.* **86,** 24903 (2015).

4. Ziabari, A. Finite Element And Imaging Approaches To Analyze Multiscale Electrothermal Phenomena. (Purdue University, 2016).

5. Cahill, D. G. Thermal conductivity of thin films: Measurements and understanding. *J. Vac. Sci. Technol. A Vacuum, Surfaces, Film.* **7,** 1259 (1989).

6. Dames, C. & Chen, G. 1w, 2w, and 3w Methods for Measurements of Thermal Properties. *Rev. Sci. Instrum.* **76,** 1–14 (2005).

7. Wilson, R. B. & Cahill, D. G. Anisotropic failure of Fourier theory in time-domain thermoreflectance experiments. *Nat. Commun.* **5,** 5075 (2014).

8. Hua, C., Chen, X., Ravichandran, N. K. & Minnich, A. J. Experimental metrology to obtain thermal phonon transmission coefficients at solid interfaces. *Phys. Rev. B* 1–47 (2016).

9. Minnich, A. J. *et al.* Thermal conductivity spectroscopy technique to measure phonon mean free paths. *Phys. Rev. Lett.* **107,** 1–4 (2011).

10. Siemens, M. E. *et al.* Quasi-Ballistic thermal transport from nanoscale interfaces observed using ultrafast coherent soft X-ray beams. *Nat. Mater. Lett.* **9,** 26–30 (2010).

11. Vermeersch, B. Compact stochastic models for multidimensional quasiballistic thermal transport. *J. Appl. Phys.* **120,** (2016).

12. Hua, C. & Minnich, A. J. Transport regimes in quasiballistic heat conduction. *Phys. Rev. B - Condens. Matter Mater. Phys.* **89,** (2014).

13. Hua, C. & Minnich, A. J. Analytical Green's function of the multidimensional frequency-dependent phonon Boltzmann equation. *Phys. Rev. B - Condens. Matter Mater. Phys.* **90,** 1–7 (2014).
